# Assessing the State of Substitution Models Describing Noncoding RNA Evolution

**DOI:** 10.1093/gbe/evt206

**Published:** 2014-01-03

**Authors:** James E. Allen, Simon Whelan

**Affiliations:** ^1^Faculty of Life Sciences, University of Manchester, Manchester, United Kingdom; ^2^Evolutionary Biology, Evolutionary Biology Centre, Uppsala University, Sweden

**Keywords:** RNA, phylogenetics, substitution model, hypothesis tests, model selection

## Abstract

Phylogenetic inference is widely used to investigate the relationships between homologous sequences. RNA molecules have played a key role in these studies because they are present throughout life and tend to evolve slowly. Phylogenetic inference has been shown to be dependent on the substitution model used. A wide range of models have been developed to describe RNA evolution, either with 16 states describing all possible canonical base pairs or with 7 states where the 10 mismatched nucleotides are reduced to a single state. Formal model selection has become a standard practice for choosing an inferential model and works well for comparing models of a specific type, such as comparisons within nucleotide models or within amino acid models. Model selection cannot function across different sized state spaces because the likelihoods are conditioned on different data. Here, we introduce statistical state-space projection methods that allow the direct comparison of likelihoods between nucleotide models and 7-state and 16-state RNA models. To demonstrate the general applicability of our new methods, we extract 287 RNA families from genomic alignments and perform model selection. We find that in 281/287 families, RNA models are selected in preference to nucleotide models, with simple 7-state RNA models selected for more conserved families with shorter stems and more complex 16-state RNA models selected for more divergent families with longer stems. Other factors, such as the function of the RNA molecule or the GC-content, have limited impact on model selection. Our models and model selection methods are freely available in the open-source PHASE 3.0 software.

## Introduction

Understanding the evolutionary relationships between species, genes, and populations is important in many areas of biology. This insight is usually obtained through the inference of a phylogenetic tree from a set of aligned sequences. The landmark article by Woese and Fox (1977) demonstrated that the presence of ribosomal RNA (rRNA) in all living organisms and its high degree of conservation make it an excellent gene for studying species relationships. Since then, rRNA has been a popular choice for phylogenetic inference, ranging from the algae that live on sloth fur (Suutari et al. 2010) to 200 metazoan species (Mallatt et al. 2010). The biological importance of rRNA (and tRNA) is well established, but recently the significance of other types of noncoding RNA (ncRNA) has been recognized (reviewed in Griffiths-Jones 2007; Mattick 2009). For these genes, phylogenetic tree estimates can be used to investigate relationships within and between families of ncRNA, in order to better understand their evolution and function. For example, a microRNA precursor might be subject to several, potentially antagonistic, evolutionary constraints, whereby the functional site(s) of the microRNA could be derived from one or both sides of the base-paired stem region (Berezikov 2011).

Inferring trees from alignments of sequences necessitates a reliable method of inference, such as maximum likelihood (ML) or Bayesian inference (reviewed in Yang and Rannala 2012). These methods require an explicit description of how sequences change over time, in the form of a parameterized probabilistic substitution model. Substitution models describing nucleotide evolution typically assume that sites in an alignment evolve independently from one another, but this assumption is difficult to justify for RNA genes where there are strong functional constraints induced by complementary base pairing in stem regions. To account for these dependencies, evolution of RNA stems is frequently described by dinucleotide substitution models, summarized by Savill et al. (2001). The earliest RNA models describe changes between 16 states, representing all 16 possible dinucleotides (Schöniger and von Haeseler 1994; Muse 1995). Later simplifications merge the ten dinucleotides representing unstable base pairs into a single “mismatch” state, resulting in models with seven states (Tillier and Collins 1998; Higgs 2000). Since their inception, there have been a wide variety of 16- and 7-state RNA substitution models, each reflecting different biologically informed descriptions of RNA evolution.

In order to investigate the improvement of RNA models over their nucleotide-based counterparts and the relative importance of their biological parameters, statistical methodology for comparing models is required. It is routine in phylogenetics for researchers to use formal model selection to decide which substitution model to use when inferring phylogenetic trees from nucleotide or amino acid sequence data (Posada and Buckley 2004; Posada 2008; Darriba et al. 2012). Common model selection methods include likelihood ratio tests for nested models and, more generally, information theoretic measures, such as Akaike's information criterion (AIC) and Bayesian information criterion (BIC) (Burnham and Anderson 2002; Sullivan and Joyce 2005). Such approaches are not appropriate for comparing models with different state spaces, such as comparisons between 4-state nucleotide models and 7-state RNA models or between 7-state RNA models and 16-state RNA models. When the models to be compared have different state spaces, it changes the data on which the likelihood calculations are conditioned (Burnham and Anderson 2002). To overcome this problem, previous studies developing RNA models have used model selection methods based on complex and time-consuming simulations (Schöniger and von Haeseler 1999; Gibson et al. 2005; Telford et al. 2005) or have avoided direct model comparisons by evaluating the recovery of a “true” tree by each model (Letsch and Kjer 2011). The majority of these studies conclude that RNA models better describe the evolution of RNA stems than nucleotide models, albeit the evidence come from a single alignment of rRNA (Schöniger and von Haeseler 1994; Rzhetsky 1995; Tillier and Collins 1998; Savill et al. 2001; Telford et al. 2005).

Here, we seek to build on previous studies investigating RNA evolution in three key ways. First, we investigate the fit of RNA models on large numbers of mammalian RNA genes derived from genomic alignments, including many different types of ncRNA. This approach provides a generalized view of the relative fit of RNA models and their applicability to large-scale genomic comparisons. Second, we develop a new method for comparing RNA models with different state spaces, based on methods created for comparing amino acid and codon models (Seo and Kishino 2008, 2009). This approach enables rapid comparisons between all RNA and nucleotide models, allowing large-scale comparison without time-consuming simulation. Third, we examine whether the choice of best-fit model affects the phylogenetic tree estimate, under the expectation that better-fitting models should provide more accurate estimates. This study finds that RNA models very frequently provide a better fit than nucleotide models across all RNA gene families, with similar patterns of model fit observed for all types of RNA. Of the different types of RNA model, we find that models describing general base pair stability, rather than the precise identity of base pairs, tend to provide a better fit than other RNA models. We also demonstrate that the choice of model can have a substantial effect on the tree estimate, with the greatest differences being between nucleotide and RNA models, but there is also substantial variation within the different types of RNA model.

## Materials and Methods

### Substitution Models

#### Definitions

In all of the models that we use, changes between states are described by a time-reversible Markov process, with rate matrix 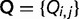, where 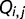 is the substitution rate between states 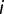 and 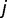 (Yang 2006). The equilibrium frequency of states is denoted by 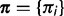, where 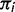 is the frequency of state 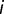. The constraint of reversibility enforces 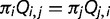 and allows 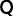 to be represented as 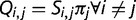, where 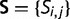 is a symmetric matrix of exchangeability parameters (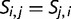), which describes the relative rate of change between 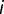 and 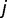. To calculate the likelihood of a model with parameters 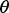 for data 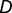, 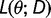, requires the creation of a transition matrix from the instantaneous rate matrix by 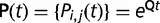, which describes the probability of change between states 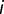 and 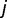 over a branch of length of 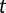, where 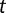 is in units of the expected number of substitutions per site. We use numerical superscripts to denote the dimension of a matrix and any values derived from that matrix; for example, 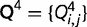 denotes a 4-state instantaneous rate matrix (Yang 2006).

#### Nucleotide and Dinucleotide Models

This study examines 18 different parameterizations of 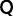 to define “foundation models” of nucleotide and dinucleotide evolution, which are later combined to provide a range of substitution models describing RNA evolution (discussed later). To describe the evolution of independent nucleotides, we use two common (4-state) foundation models: 1) the HKY model (Hasegawa et al. 1985) and 2) the general time-reversible (GTR) model (Lanave et al. 1984; Tavaré 1986). Both nucleotide foundation models are always used in conjunction with Г-distributed rates-across-sites, indicated by a “+Г” suffix (Yang 1994). To describe evolution in base pairs, we examine a range of foundation models over two different state spaces: 1) 16-state foundation models describing substitutions between all possible base pairs and 2) 7-state foundation models describing substitutions between the six stable canonical base pairs (the Watson–Crick base pairs A:U and C:G and the “wobble” pairing G:U) and a mismatch state, which contains the ten other base pairs (A:C, A:G, C:U, A:A, C:C, G:G, and U:U). Following the naming convention of Savill et al. (2001), we investigate nine 16-state dinucleotide foundation models (16A, 16B, 16C, 16D, 16E, 16F, 16I, 16J, and 16K) and seven 7-state dinucleotide foundation models (7A, 7B, 7C, 7D, 7E, 7F, and 7G). The parameterizations and original authorship of these models is given in the PHASE 3.0 manual. All models have previously been described except 7G, which we propose here as a natural simplification of 7E and 7F. Under 7G, the instantaneous rate matrix is defined as:
(1)
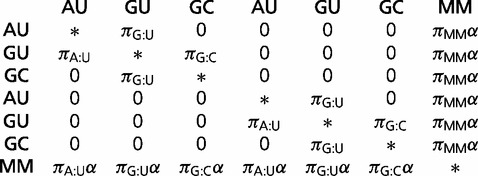

where 
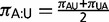
, 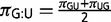, 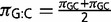, and 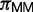 is the total frequency of the mismatch states. We do not examine the early 6-state models, such as those proposed by Tillier and Collins (1995), because with modern computing power it is unreasonable to recode unstable base pairs as missing data, rather than explicitly incorporate them into the model.

**Fig. 1.—:**
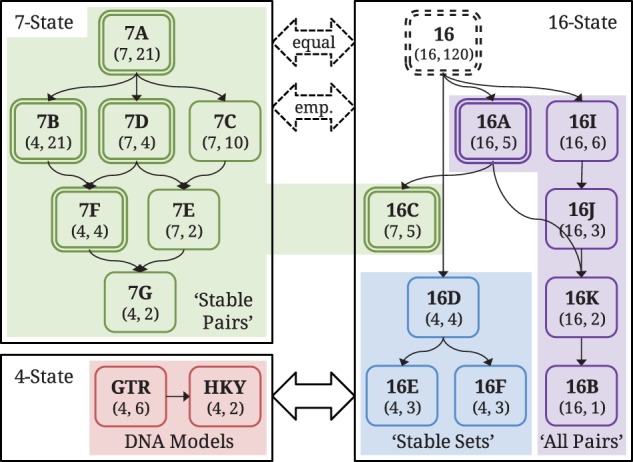
Summary of the parameterization of RNA and DNA models and the relationships between them. The values below each model name are the number of frequency and exchangeability parameters, respectively. Double borders around models indicate that double substitutions are permitted. Arrows between models indicate nesting. The general 16-state model (dotted box) has too many parameters to be tractable and is not included in this analysis. The 4-state and 16-state models are directly comparable. The 7-state models require a likelihood adjustment value to account for the mapping from 1 mismatch state to 10, which can use either equal frequencies (0 degrees of freedom) or empirical frequencies (9 df).

Figure 1 shows a summary of the parameterization of the 18 foundation models described above, the relationships between them, and how they can be grouped into four classes depending on how they deal with paired bases. The first class (red), consisting of HKY + Г and GTR + Г ignores base pairing and allows nucleotides to evolve independently. The remaining three classes are determined by how they describe the selective pressures acting on dinucleotides, primarily defined by the parameterization of 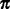. The foundation models contained in the “All Pairs” class (purple) consider changes between the 16 possible dinucleotides, allowing each dinucleotide, XY, to have its own equilibrium frequency, 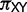. The “Stable Pairs” class (green) has models with separate frequencies for each of the stable base pairs (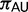, 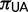, 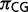, 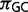, 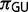, 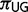) and groups the ten mismatch base pairs together into a single frequency parameter (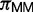). This restriction is simple in 7-state dinucleotide models where each state has its own frequency, whereas dinucleotide frequencies for the ten mismatch states in 16C are defined as 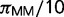. Note that models 7B, 7F, and 7G place the further restriction of strand symmetry, resulting in three frequencies for the stable base pairs (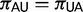, 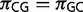, and 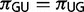) and a single frequency describing mismatches (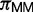). Finally, the “Stable Set” foundation models (blue) define their equilibrium frequencies based on the product of the individual nucleotide frequencies and two parameters describing the tendency for stable base pairs to occur (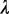) and for wobble pairings to occur (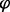). In these foundation models, the equilibrium frequency of the dinucleotide XY is given by 1) 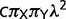 for Watson–Crick base pairs; 2) 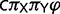 for wobble base pairs; and 3) 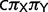 for mismatch base pairs, where 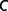 is a scaling constant. Note that the instantaneous rate of change between dinucleotides for the Stable Set is different to the other two classes because its parameters adjust both the substitution rates between dinucleotides and the equilibrium frequency of those nucleotides (for full details of all dinucleotide models, see the PHASE 3.0 manual and Savill et al. 2001).

#### Modelling RNA Evolution

The foundation models described above are combined by a fixed-effect mixture model to create RNA substitution models, where partitions are specified a priori. The loop regions of the RNA are specified in the alignment and may be modeled by either of the two single nucleotide foundation models (HKY + Г or GTR + Г). The base-paired stems may be modeled by either of the 2 single nucleotide foundation models or by any of the 16 dinucleotide foundation models with or without Г-distributed rates-across-sites, yielding 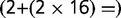 34 possible stem models. The different combinations of stem and loop foundation models produces 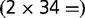 68 mixture models. A further two, non-mixture, models are also used, in which a single nucleotide model (HKY + Г or GTR + Г) is used, ignoring the loop and stem partitions. For models where the loops and stems are partitioned, we also incorporate a scaling factor, 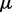, describing the evolutionary rate of stems relative to that of loops. This scaling factor can then be used to calculate meaningful evolutionary rate for the RNA gene, 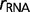, in terms of 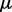, the unconstrained rate of nucleotide evolution, 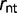, and the probabilities of a nucleotide belonging to a stem or loop, 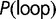 and 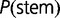, such that 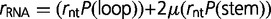. Note that the “2” is required because RNA dinucleotide models are usually scaled to change at one substitution per dinucleotide per unit time. This relationship allows simple comparison between tree lengths obtained under different models.

### Model Comparison

To compare the different RNA substitution models, we use the corrected version of AIC (Akaike 1974; Burnham and Anderson 2002): 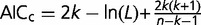, where *k* is the number of parameters, *L* is the likelihood, and *n* is the sample size. An approximation to the sample size is computed by counting the characters in an alignment, treating each base pair as a single character in the case of RNA models, following the approach of Posada and Buckley (2004). Standard likelihood theory demonstrates that it is not valid to compare likelihoods computed in different state spaces, preventing the simple comparison of AIC_C_ values of models with different state spaces (Burnham and Anderson 2002). In other words, it is not possible to compare between the groups of 4-state DNA models, 7-state RNA models, and 16-state RNA models. Previous research has used sophisticated simulation schemes to compare models (Savill et al. 2001; Telford et al. 2005). Instead, we use an approach that projects 4-state and 7-state models to a 16-state space, which provides valid likelihood comparisons. This technique has been previously described for transforming DNA, amino acid, and codon models into 64-state models (Whelan and Goldman 2004; Seo and Kishino 2008, 2009). We extend these authors' work for the comparison of DNA and RNA models, highlighting the required modifications of their mathematical proofs.

#### Comparing 4-State and 16-State Models

Previous research has shown that 4-state nucleotide models and 64-state codon models are directly comparable (Whelan and Goldman 2004). In order to show that 4-state nucleotide and 16-state dinucleotide models are directly comparable, we follow closely the proof of Seo and Kishino (2008, 2009). We observe that a dinucleotide model in which one nucleotide is fixed is equivalent to a 4-state model for the unfixed nucleotide:
(2)
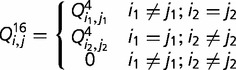

where 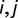 are dinucleotides and 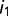 and 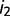 are the nucleotides at the first and second position of the dinucleotide, respectively. (Note the diagonal entries of all 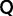 matrices are defined by the constraint that the row sum is 0.) The matrix 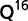 derived from equation (2) can be decomposed into two matrices, 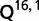 and 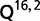, which describe the transition rates of the first and second nucleotide, respectively. These two matrices are commutative, so 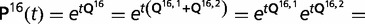 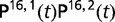.

The rows (or columns) of any 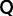 matrix can be interchanged without affecting the validity of the matrix, allowing the rearrangement of the rows and columns of 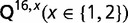 to obtain “diagonal block” matrices which have 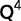 on the diagonal and zeroes elsewhere. The calculation of 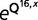 is then equivalent to a diagonal block matrix with 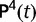 on the diagonals, and the rows and columns of 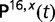 can subsequently be rearranged to restore their original order. Finally, the product 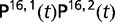 gives the original matrix 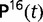 leading to
(3)
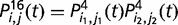



Following the proof of equation (11) provided in the appendix of Seo and Kishino (2009), it is possible to derive 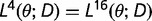 using our equation (3) and demonstrate that the likelihoods of 4-state and 16-state models are directly comparable.

#### Comparing 7-State and 16-State Models

The likelihoods of 7-state and 16-state models cannot be directly compared, but one can devise a likelihood correction value that corresponds to projecting the 7-state model to 16-state space. We note that it is also possible to transform a 16-state model to 7-state space, but as this is of limited practical use we do not describe such a mapping; it is a simpler version of the conversion from a codon to an amino acid model given in Yang et al. (1998). The transformation from 7-state to 16-state follows that in Seo and Kishino (2008), in which a mapping was defined from a 20-state amino acid model to a 61-state codon model. We define the off-diagonal values of a 16-state matrix in terms of parameters from a 7-state matrix:
(4)
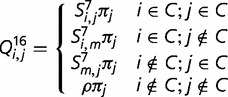

where 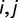 are dinucleotides, 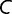 is the set of canonical dinucleotides, and 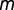 is the compound mismatch state in the 7-state model. The substitution rate between mismatches is undefined in the 7-state model, so in the 16-state model we define it in terms of the dinucleotide frequency, 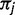, and a new exchangeability parameter, 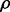, which describes the rate that mismatch dinucleotides substitute one another.

Following the work of Seo and Kishino (2008), it is possible to optimize 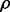, which would create a new class of RNA models that lie somewhere between 7-state and 16-state models. We do not investigate this possibility here, however, because the rate of change between mismatches is of limited interest and including it would introduce a large number of additional models to our analysis. We also note that the 16C model is extremely similar to such a model, because it was created as an extension of model 7D. Instead, we concentrate on making existing 7-state models comparable with 16-state models. First, we assume that 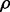 in equation (4) is infinite. This parameterization, discussed in detail by Seo and Kishino (2008), makes all mismatch states in the 16-state model equivalent because all 10 states instantaneously reach the same equilibrium distribution. The parameterization also means that all directly comparable substitution rates are the same for the 7-state and the transformed 16-state model, including the overall rate of substitution back and forth between matches and mismatches. Therefore, the original and transformed models are equivalent and differ only by the state space that they are conditioned upon. Next, we need a likelihood “correction” to account for the different state spaces, which is obtained following the proof of equation (6) in Seo and Kishino (2008) to obtain:
(5)
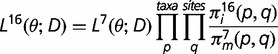

where 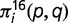 is the frequency of the 16-state dinucleotide at position 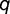 in taxa 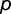, and 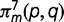 is the frequency of the 7-state dinucleotide at position 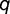 in taxa 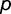. For match states this ratio is 1, whereas for mismatch states it is the frequency of the specific mismatch dinucleotide in the 16-state model divided by the sum of the frequencies of mismatch states. Projecting the single mismatch state of the 7-state models into ten distinct states means that each of the frequencies needs to be defined. We apply this projection to AIC_C_ calculations in two different ways: 1) assuming that all noncanonical dinucleotides are equally likely, so that 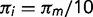; and 2) using empirical frequencies. The former approach is equivalent to an unparameterized model with no prior knowledge of (di)nucleotide frequencies, whereas the latter is the equivalent of taking ML estimates of the nine additional parameters introduced in equation (5). For each 7-state model, we compute likelihoods for both projections and choose the one that provides the lowest AIC_C_ for full model comparison.

### Implementation and Tree Search

All phylogenetic analyses are performed with a modified version of the PHASE 2.0 software package (Hudelot et al. 2003; Telford et al. 2005; Gowri-Shankar and Rattray 2006), which we call PHASE 3.0. Open-source software, full instructions on program usage, and an updated manual are available at https://code.google.com/p/rna-phase-3/ (last accessed January 3, 2014). Further to the addition of the 7G dinucleotide model and state-space projection, PHASE 3.0 also includes several bug fixes and updates, leading to improved program stability and accuracy. All model comparisons are performed under ML on a fixed tree topology, which is estimated with the bionj algorithm (Gascuel 1997) implemented in phyml (Guindon et al. 2010) that uses a model of the variance and covariance of evolutionary distances. Phylogenetic tree search is performed using Bayesian MCMC analysis to obtain samples from the posterior distribution across all parameters, including trees, branch lengths, and model parameters. The results from the ML inference are used as the starting point for the MCMC, followed by 150,000 burn-in iterations. In total 300,000 sampling iterations are performed, with a sampling period of 100, yielding 3,000 posterior samples. Under ML and Bayesian inference, the (di)nucleotide frequency estimates are obtained from empirical counts from the sequence data, with no subsequent optimization.

### Genomic Alignments of RNA Genes

We extract all human RNA sequences from the alignments in the Rfam “seed” data set (version 10.1), a total of 1,255 distinct sequences, associated with 550 Rfam families (Gardner et al. 2011). We also extract structures from Rfam and discard the 194 sequences which have a gap at a position that corresponds to a paired base in the structure, as gaps are subsequently removed from the sequences and the structures would then become invalid. The remaining 1,061 sequences are mapped to the human genome (GRCh37/hg19) using a BLAT search (Kent 2002) to identify perfect matches. We reject sequences that return no hits or that map to discontiguous genome sequence. The BLAT result for each sequence provides a genomic location; if there are several locations with the same BLAT score, we discard all of those locations. Locations on the mitochondrial genome are also ignored; and if two locations overlap, both are discarded. These filtering steps result in 858 distinct genomic locations corresponding to members of 480 Rfam families.

Rfam provides alignments of the RNA sequences in a family, but these are built with reference to their secondary structure, rather than the evolutionary history of any particular locus. As we are interested in the latter, we retrieve the EPO-12 and EPO-35 mammalian genomic alignments from Ensembl (Paten et al. 2008; Paten et al. 2009; Flicek et al. 2012), which are estimated using the EPO genomic alignment pipeline for 12 and 35 mammalian species, respectively. To ensure the genomic alignment procedure does not bias our results, we also compare results obtained using the Multiz alignment tool for the 11 species shared with EPO-12 (pig is not present in Multiz). A wide range of quality control checks were performed on these alignments, including removing those with 1) multiple genomic blocks or inadequate flanking sequence; 2) ambiguous bases; 3) long insertions or deletions in the human sequence; 4) fewer than five sequences; 5) evidence for gene gain/loss; 6) overlap with an annotated mRNA; and 7) poor fitting RNA structure, assessed by a Structure Conservation Index (SCI) <0.8 (Gruber et al. 2008). After these filters, the final data sets consist of 287 alignments covering 203 RNA families for EPO-35, 124 alignments covering 107 RNA families for EPO-12, and 182 alignments covering 149 families for Multiz. Many of these alignments were also manually examined, but not edited, to ensure consistent quality throughout. All the alignments are available as a zipped file on the PHASE 3.0 website: https://code.google.com/p/rna-phase-3/ (last accessed January 4, 2014).

## Results

The results for the three different sets of genomic alignment (EPO-12; EPO-35; Multiz) show very similar patterns, so for brevity we present only those obtained from the EPO-35 alignments as these provide the largest and most comprehensive data set. Results from the other alignments are available from the authors upon request.

### Dinucleotide Substitution Models Better Describe RNA Evolution

Table 1 shows the best-fitting model for the 287 RNA gene alignments in the EPO-35 data set. The substitution process in nearly all of the alignments (281/287 = 98%) is best described by an RNA model that describes dinucleotide evolution in the stem region explicitly. Two models best describe evolution in over half of the alignments, our new 7G model, the simplest of the Stable Pairs set, and the most complex Stable Sets model, 16D. The 7G model is, indeed, the simplest of all RNA models (fig. 1) with only four free parameters (eq. 1) and tends to be selected in the most conserved alignments. The rarely selected HKY model has the same number of parameters as 7G, suggesting that even when there are relatively few changes in an alignment, then an RNA model provides a better description of those changes and the relative nucleotide frequencies than a DNA model. When a 7-state model is selected, it is almost always (78/80 = 98%) the variant that uses equal, rather than empirical, mismatch frequencies for the correction that projects the likelihood onto a 16-character state-space.

**Table 1:** Number of Best-Fit Substitution Models for EPO-35 RNA Genes

Model Class	Loop Model		
Stem Model	HKY + Γ	GTR + Γ	Total
DNA			
One DNA model	6	0	6
Two DNA models	0	0	0
Stable Pairs			
16C	18	5	23
7C	4	0	4
7E	7	1	8
7F	1	0	1
7G	58	9	67
Stable Sets			
16D	93	27	120
16E	33	8	41
16F	12	2	14
All Pairs	2	1	3
Total	234	53	287

In the few cases where a DNA model is selected, it is always a single model covering loop and stem, rather than a model partitioned for stems and loops. In the 281 alignments where an RNA model is chosen, the loop regions are best described by the simpler HKY + Γ, rather than GTR + Γ, in 234 (83%) alignments. In addition to the information shown in table 1, we find the best-fit RNA models rarely include rates-across-sites heterogeneity, with only 14% of alignments using a +Г dinucleotide model, suggesting that all base pairs in a stem tend to evolve at a similar rate. This observation notably contrasts with the tendency for nucleotide (Arbiza et al. 2011) and amino acid (Goldman and Whelan 2002) alignments to provide significant support for spatial rate heterogeneity.

**Fig. 2.—:**
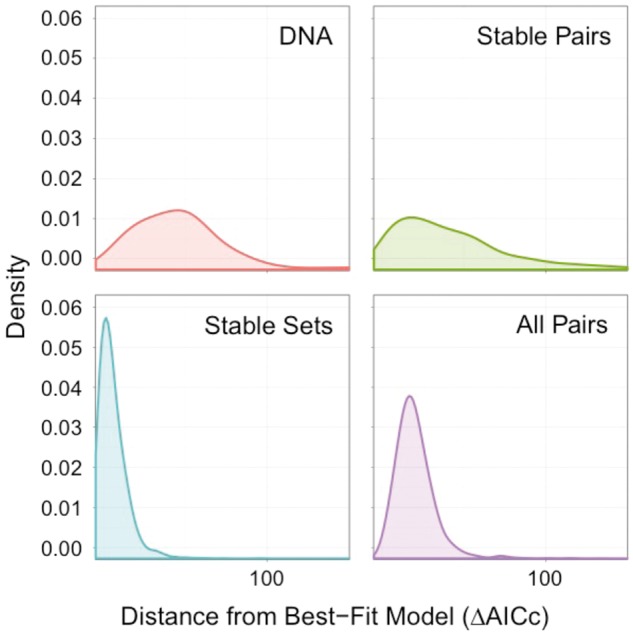
Distribution of ΔAIC_c_ values relative to the best-fit model (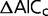), calculated across all models. Models with 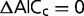 are not included. Note that the *x* axis is truncated at 150 for clarity.

Simply examining the best-fit model may be misleading, because when there are several similarly fitting models small differences in the likelihood may lead to different models being chosen. Figure 2 shows the distribution of AIC_C_ values for each class relative to the best model. In cases where the Stable Sets models are not selected as the best models, their AIC_C_ values tend to be very close to those of the best-fitting model, suggesting that they consistently provide a good fit to the data even if they are not the absolute best model. The Stable Pairs class is much more inconsistent; in some cases it fits well, but in others it fits very poorly. Although 7G is often chosen as the best fit model, in the remaining cases it does not fit as well as the Stable Sets models, especially 16D, which is the first or second choice model for 242 (85%) RNA gene alignments.

The parameter estimates obtained from the dinucleotide foundation models provide some insight into RNA function and evolution. The empirical frequency of Watson–Crick base pairs is 80%, with the remaining base pairs consisting primarily of wobble base pairs (13%) and a smaller proportion of mismatches (7%). These frequency estimates are used directly by the Stable Sets models through the influence of the 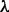 and 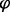 parameters, which control the relative frequency of, and the substitution rates between, the Watson–Crick, wobble, and mismatch dinucleotide pairs. In contrast, the Stable Pairs models do not differentiate wobble mismatches from other mismatches and lump both together into a single mismatch (MM) category. Therefore, the relatively strong preference for 16D over other models suggests that different selective pressures act on wobble and other mismatches, and for some types of RNA it is important to differentiate between them when parameterizing a dinucleotide model.

The frequency estimates and the best-fit models both demonstrate, as expected, that there is consistent and strong evidence for stable stems, and that wobble pair pairing is a viable intermediate during RNA evolution. Although mismatches do occur, albeit relatively infrequently, the very low frequency (1%) with which All Pairs models are chosen suggests that the exact identity of mismatches when they occur is unimportant. Examining the relative rate of per nucleotide substitution in loops and stems, 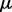, just under half of the RNA genes (49%) have a faster rate in stems than in loops. In many cases, the difference is small, but 21% of the RNA genes have a stem rate over twice that of the loop rate.

### Factors Determining Model Choice

It is of interest to understand the factors affecting model choice as these may aid identification of novel RNA genes or the classification of existing genes. The type of RNA gene has some effect on model choice (table 2), but in cases where there is more than one example of an RNA type, no single class of models is exclusively chosen. Rather than having a direct relationship with the type of RNA gene, model choice appears more closely related to the amount of structural and evolutionary information available. In the few cases where they are selected, the DNA models mostly describe evolution in snoRNA that have relatively few base pairs.

**Table 2:** Best-Fit Models for EPO-35 Alignments, Classified by RNA Type

RNA Type	Model Class			
	DNA	Stable Pairs	Stable Sets	All Pairs
Long ncRNA	0	9	15	0
microRNA	0	33	71	2
Ribosomal	0	0	1	0
RNase P	0	1	0	0
scaRNA	0	2	9	0
snoRNA	4	52	62	1
Spiceosomal	0	1	0	0
tRNA	0	1	0	0
Vault	0	0	1	0
Other^a^	2	4	16	0

^a^A heterogeneous mixture of molecules such as cis-regulatory elements and selenocysteine insertion sequences that do not naturally fit into other groups.

**Fig. 3.—:**
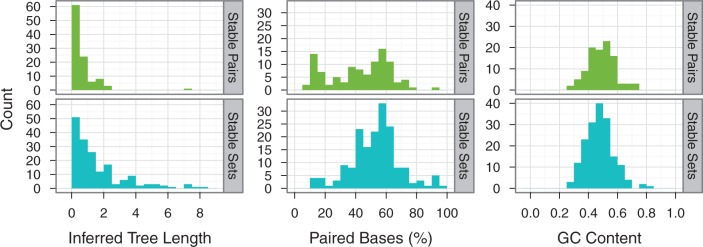
Factors affecting model choice for the Stable Pairs and Stable Sets models: GC content, percentage of paired bases, and inferred tree length, where tree length is the sum of the individual branch lengths under the best-fit model.

Figure 3 shows various factors that previous studies have suggested are important to RNA evolution. Tree length measures the total number of evolutionary events in an alignment. When few events occur, the Stable Pairs models tend to be selected most often. As greater numbers of substitutions are inferred, on larger numbers of paired bases, the Stable Sets models tend to dominate. Factors such as GC content and the number of gaps in an alignment (not shown) do not lead to a preference for one category of model over another.

### The Effect of Model Choice on Tree Inference

**Fig. 4.—:**
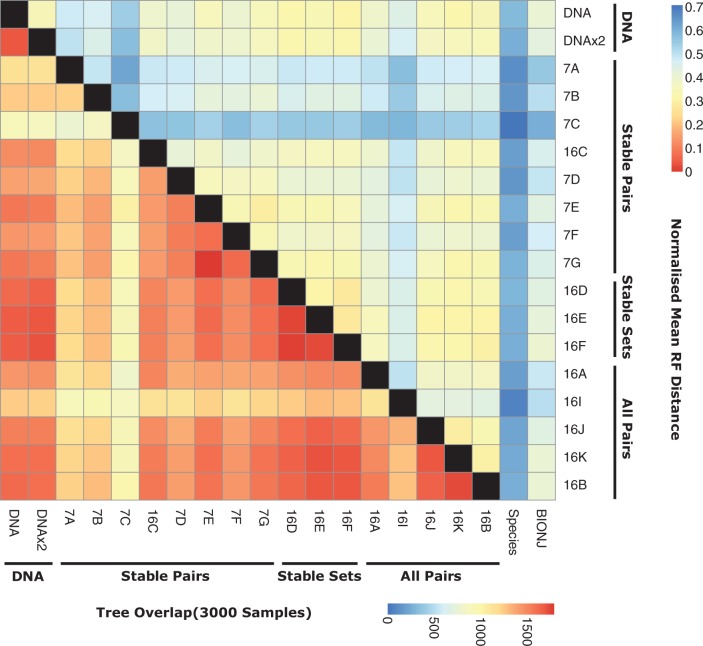
Effect of model choice on tree inference. Rows and columns in the heatmap represent different models. Data are shown for HKY loop models and -Γ dinucleotide models. Within a class, the models are listed in an order that approximates decreasing model complexity, from left to right (top to bottom). The lower left off-diagonal shows the mean overlap between the 3,000 sampled trees from each MCMC tree search. The upper right off-diagonal shows the mean Robinson–Foulds (RF) distance between sampled trees, normalized by the number of branches in the tree so that alignments with different numbers of taxa are comparable. The trees estimated from RNA models are also compared to the EPO-35 species tree and a neighbor-joining bionj tree.

We use a Bayesian inference approach for studying the effect of model choice on tree inference. For each RNA gene alignment, we use PHASE 3.0 to take a set of 3,000 samples from the posterior distribution of tree topologies under each of the models described in figure 4. We investigate two similarity measures to compare sets of posterior trees estimated under each of the models. The first measure, shown in the lower off-diagonal of figure 4, is the proportion of trees that overlap between the two posterior sets of trees, providing a general insight into the similarity of the cloud of trees present in both sets. The second measure, shown in the upper off-diagonal of figure 4, is the mean Robinson–Foulds (RF) distance between the posterior distribution set of trees, normalized for each pairwise comparison so that a distance of 0.0 represents identical trees and 1.0 represents no shared branches. This measure provides insight into the similarity of the trees and the variance in their estimates. Similarity because sets of trees with similar branching patterns will tend to have low average RF distances; and variance because higher variance estimates may have higher average RF distances, since the majority of random trees from large data sets tend towards having a (normalized) RF distance of 1.0 (Steel and Penny 1993).

We qualitatively summarize these results as a broad agreement between the sets of trees inferred under many of the models, with the caveat that the specific choice of model can affect the finer detail of the topology; an expected outcome as all models are trying to capture the same evolutionary tree structure. The exceptions to this broad pattern are models 7A, 7B, and 7C (and to a lesser extent models 16A and 16I) whose posterior sets of tree estimates exhibit markedly less similarity to the other models and to one another. These three (five) models tend to be the most parameter rich (see fig. 1), particularly 7A, 7B, and 7C, that have a number of parameters describing rates of substitution into and out of the mismatch state. There is, however, no clear relationship between the number of parameters and the decrease in similarity measures indicative of the increase in variance associated with high numbers of parameters. Model 7A, for example, has the highest number of parameters (28) but has more similar sets of trees to other models compared with model 7C, which has 17 parameters.

Finally, figure 4 also shows an unexpectedly large normalized RF distance between the sets of posterior tree estimates from all models and the EPO-35 “species tree” provided by Ensembl. To evaluate whether these differences are significant, we conducted an AU-test between the majority rules consensus tree from the Bayesian analysis under the best-fit model and the Ensembl species tree. We find significant differences between these trees in 124 (43%) of the alignments, suggesting that the lack of similarity is not due to sampling variance. The broad agreement between the trees estimated under the majority of the models and their similarity to trees estimated from other software, such as bionj, lead us to conclude these differences are a property of the RNA genes rather than an artifact of the software or modeling process.

## Discussion

In this study, we introduce a new and powerful set of methods for RNA model selection, allowing for the first time simple comparison between 4-state DNA models and their 7-state and 16-state RNA model counterparts. Based on related theory linking together nucleotide, amino acid, and codon models (Whelan and Goldman 2004; Seo and Kishino 2008, 2009), we project all sets of models to a 16-state space, which allows direct comparison of their likelihoods through information theoretic measures, such as AIC and BIC (Burnham and Anderson 2002). This model selection methodology complements those already available for comparing nucleotide (Posada 2008) and amino acid models (Darriba et al. 2011).

Our projection method does, however, have some limitations linked to the relationship between 7-state RNA models and their 16-state projections. Our method is based on that of Seo and Kishino (2008), who apply the same strategy to compare amino acid and codon models, and our projected model represents one of the many possible 16-state models compatible with the original 7-state model. Different values of 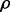 in equation (4), for example, could be used to project a series of 16-state models all compatible with the instantaneous rate matrix of the original 7-state model. In order to allow valid model comparison, we would like the process of change described by the original 7-state model to be unaffected by the projection process, which allows direct simple and direct comparison to other 16-state models. Our approach of assigning 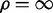 results in a projected 16-state model where the probability of change from a mismatch state to a match state is independent of the original (unobserved) mismatch. An intuitive explanation of this independence is that the mismatches substitute one another instantaneously and are therefore indistinguishable from one moment to the next. Further research is required to demonstrate that our projection method is the optimal strategy for RNA model comparison, but until then we suggest it provides a useful tool when selecting or comparing RNA models.

To demonstrate the utility of RNA dinucleotide models and the selection process we propose, we examine a large set of vertebrate RNA genes derived from human genes identified in Rfam (Gardner et al. 2011). Of the 287 RNA genes that pass our stringent filtering criteria, we find 281 genes support the selection of an RNA-specific model in preference to a DNA model. This finding supports those of other smaller scale studies that have shown the value of RNA dinucleotide models, albeit through more complex model selection criteria (Schöniger and von Haeseler 1994; Rzhetsky 1995; Tillier and Collins 1998; Savill et al. 2001; Telford et al. 2005). Our analyses demonstrate that 9 of the possible 16 dinucleotide models are supported by at least one or more genes, demonstrating the necessity of rigorous model selection. Despite the range of models available and the opportunity for fast model selection, it is of interest to know which of the RNA substitution models tends to perform best on average. By examining differences in AIC_C_ between models, we show that Stable Sets models tend to produce either the best-fit model or close to the best-fit model for the majority of RNA genes examined. If one wishes to conduct exploratory data analysis under a single RNA model, then our results suggest 16D would be a reasonable choice. Given the breadth of selected models and their effect on downstream inference, we recommend a full model selection procedure for more detailed evolutionary studies.

Our study suggests that two factors primarily affect model selection in RNA genes: the evolutionary divergence between the sequences and the number of paired bases in the RNA structure (fig. 3). These observations can be rationalized by considering what the dinucleotide component of RNA models attempts to describe. First, the relative frequency of dinucleotides in stems is biased away from the product of the constituent (single) nucleotide frequencies, which helps RNA models better describe sequences even when there is little sequence divergence. A greater number of nucleotides in stems tends to result in a concomitant improvement in fit of RNA models. Second, as sequences diverge, there is a greater opportunity for the parameters in the dinucleotide model to improve model fit by describing those changes. Improvement in model fit is therefore also related to the product of evolutionary divergence (tree length) and the number of paired bases, which describes the total number of changes observed in the RNA structure. The choice of parameters affected by increasing numbers of changes is, however, heterogeneous, evidenced by the wide range of models chosen. Other factors, such as the function of the RNA gene or the GC-content, have substantially less effect.

The choice of RNA or DNA model can also have a substantial effect on phylogenetic tree inference. The broad agreement between all models is indicative of the evolutionary history of the sequences, whereas the specific differences observed reflect variation in tree estimates induced by models and the variance of those estimates. Some models, most notably 7A, 7B, and 7C, tend to produce substantially different sets of trees compared with the other models, possibly due to their high number of parameters or variation in the structure of the substitution matrix. Surprisingly, no form of model produces trees that agree with the species tree provided by Ensembl. This observation holds despite trying a wide-range of filtering procedures and software, beyond the scope of the results presented here. These tests lead us to the tentative conclusion that the differences in tree topology are a property of the genomic alignments of RNA genes rather than the models, perhaps resulting from the inclusion of paralogous genes or complexities in the evolution of RNA genes that are not captured by any of the models examined, such as arm switching in microRNAs (Griffiths-Jones et al. 2011) or changes in RNA secondary structure of, for example, ribosomal or tRNAs (Caetano-Anollés 2002).

All of the models and model comparison methods described here are implemented in the open-source PHASE software, which will allow other users to apply our methods to their analyses of RNA genes. The results of model selection can then be carried through to phylogenetic tree inference using either PHASE or other software that implements RNA substitution models, such as RAxML. Fast and rigorous model selection and model averaging (Posada 2008) may provide more robust classifications of RNA molecules and new insights into their function.
